# A corpus-based study on the cognitive construction of security in discourse

**DOI:** 10.3389/fpsyg.2022.1069896

**Published:** 2023-01-06

**Authors:** Chajuan Hu

**Affiliations:** ^1^College of International Studies, National University of Defense Technology, Nanjing, China; ^2^College of Liberal Arts, Nanjing University of Information Science and Technology, Nanjing, China

**Keywords:** critical discourse studies (CDS), cognitive approach, corpus-based analysis, security discourse, proximization, securitization theory

## Abstract

A discourse-based approach to understanding security has been explored in the study of International Relations, yet how other agents rather than the political agents speak to conceptualize the emotive appeal in unconventional security issues is less discussed. This corpus-based cognitive critical discourse study examines security by combining the International Relations’ theory of securitization with the proximization approach in Critical Discourse Studies. As a case study, texts concerning Confucius Institutes on the National Association of Scholars’ official website from 2014 to 2020 were collected to discuss how the threat is constructed discursively and cognitively for an endeavor to influence the public and the political decision-making process. The corpus was further divided into two sub-corpora in order to expose the difference in their cognitive construction of Confucius Institutes. The findings show that the American academia delivers a bottom-up securitizing move by constructing education security discourse on Confucius Institutes in the initial process, yet later the whole-of-society security narratives interacting with a top-down securitizing move from the political agents have been adopted. As indicated by the corpus statistics, the concerned discourses are discursively constructed by following the “Self-Other” dichotomy security narratives, in which Confucius Institutes are cognitively transformed from an academic issue to a national security issue and legitimized through proximization in the spatial, temporal, and axiological dimensions.

## 1. Introduction

Security discourse is produced in the political sphere of late industrial societies by national governments, along with their agencies and satellite organizations, and then is reproduced within the public sphere through multiple forms of press and media (Krzyżanowski and Tucker, 2018). When it comes to US security discourses, the 9/11 terrorist attacks had a dramatic impact on the US security agenda and its discursive constitution. After the delivery of the *9/11 Commission Report*, a “state of emergency,” or as Agamben (2005) puts it “a state of exception,” has been invoked to normalize the curtailment of civil liberties and suspension of citizenship rights. Martin and Simon (2008) also argue that US Department of Homeland Security documents maintains “a state of exception” through the discursive construction and maintenance of continuous threat. In line with the discursive articulation of temporal and typographical dimensions in constructing future disasters, Dunmire (2011) traces the legitimation of the doctrine of pre-emptive action through the realization of the future “threat” in US National Security Strategy documents and presidential speeches. In her later studies, she also argues how US security discourse provides the rationale for an expansionist security strategy that focuses on shaping global society in ways that accord with US values and interests (Dunmire, 2015). Recent studies also argue that the resonance of US security narratives lies in the way conceptualizing emotive appeal that creates perceptions of enmity and threat through their dualistic structure (Van Rythoven, 2015; Amin, 2019; Berrocal, 2019; MacDonald and Hunter, 2019; Homolar, 2021). Moreover, there have been many scholarships addressing the US security issues from an ideational perspective. These studies examine the ideological, rhetorical, and linguistic features of US political and media security discourses, highlighting that the discourse system is mainly construed through presenting a series of assertions by creating the Self-Other dichotomy for their preferred interpretations of the presented representations (Miller and Rose, 2008; Amin, 2019; Berrocal, 2019; Homolar, 2021). A focus on the split of the international arena into two opposing spheres to convey understandings of security is not new in itself (Campbell, 1998; Neumann, 1999; Said, 2003), while the nexus between agents’ discursive practices and the affective process has recently gained traction across the disciplinary field of International Relations (IR) (Solomon, 2014; Åhäll and Gregory, 2015; Koschut et al., 2017; Brassett, 2018; Hall and Ross, 2019).

The aforementioned studies provide a critical entry point for a discourse-based approach to analyze and interpret complex securitizing processes, yet how the academic agents rather than the political and the media agents “speak” to conceptualize the emotive appeal in specific unconventional security issue, i.e., education, is not fully discussed yet. Therefore, this study contributes in the following ways. First, this paper concentrates on what the academic agents in the United States speak to conceptualize and legitimate the appeal to take exceptional political actions toward Confucius Institutes (CIs), which could be helpful to show a full picture of the complex securitizing process when it comes to an education security issue. Besides, this critical discourse case analysis attempts to explore the theoretical and empirical evidence that security discourse analysis could be more demonstrative when language features and textual properties of discourse are explained in light of theories of cross disciplines, for instance, a combining of theories in the field of IR and Critical Discourse Studies (CDS) is employed in this study. Furthermore, given that methods are criticized as limited in securitization studies, a corpus-based cognitive approach to investigate the intersubjective meaning-making process in a particular securitization case would be a new methodological practice to extend the universality of securitization theory.

Drawing specifically on US education security rhetoric as an empirical anchor, the point of departure for this study is to integrate concepts of securitization theory with cognitive analytic methods of CDS to demonstrate the need for inter-disciplinary research into identifying the cognitive dimensions of the language across different genres in the study of security discourse. This paper is then divided into three sections. The first section introduces the theoretical framework, in which a cognitive analytical framework with cross-disciplinary concepts and methods for security discourse is proposed. The second section conducts a corpus-based case study of security discourse toward Confucius Institutes by the American National Association of Scholars (NAS), an academic group actively pushing for the closure of CIs. The empirical analysis is centered on texts from 2014 to 2020, which is defined as the cognitive transformation period according to the critical socio-political events listed in this section. Employing the framework proposed in the previous section, the ways in which US academic agents speak to conceptualize Confucius Institutes are discussed based on the corpus statistics. The final section draws some conclusions on the proposed analytic framework in terms of its application in education security discourses as well as its implications in IR studies of securitization.

## 2. Theoretical framework

### 2.1. Securitization and the discourse

Securitization theory has become one of the most frequently used approaches in security studies since it was fully developed in 1998. Buzan et al. (1998) define securitization as an extreme version of politicization. According to the theory, issues are prioritized and constructed as security threats *via* speech acts whereby the securitizing actor convinces the audience that the given issue is an existential threat to a referent object that must be protected. The Copenhagen School adopts a constructivist ontology and argues that security issues are intersubjectively constructed (Buzan et al., 1998). In line with this, the most crucial aspect of the construction of security issues is the discursive construction of elements that together form a securitizing move. The theory presents analyzing units for the analysis, i.e., securitizing actor, functional actors, referent object, existential threat, and the audience, and provides a clear path by examining the speech acts of the securitizing actor to identify how they convince their audience that a given issue is an existential threat to the referent object. With the help of the framework, analysts can determine how different issues are constructed as security issues through speech acts of securitizing actors. More than focusing on the magic power of speech acts, securitization theory later has gone through a rich theoretical development. Paris School scholars emphasize, with a bottom-up framework, the institutionalizations and routinizations through repetitions of security practices that produce security issues (Balzacq, 2005, 2011; Rothe, 2016). With considerations of bottom-up characteristics of the process, securitization is a dynamic, non-linear process over time in which the role of the audience is equally specified and fully considered. As Baysal (2020) argues that securitization process includes the definition of security, discursive efforts to convince the audience, and security practices that normalize and routinize the security definition. In light of the above studies, the study regards securitization as a process of truth production, especially a threat construction as the key part, behind which there are always interests and relations of power, and the discursive practice itself works as a dynamic over-time process in which multi-interactive securitizing moves take place from both a top-down and bottom-up approach.

### 2.2. Cognitive critical discourse approach: Proximization

As Chilton (2004, 2014) posits that people possess a mental ability to structure their cognitive experience by looking at the world in terms of dichotomous representations of good and evil, right, and wrong, acceptable and unacceptable, etc., and this ability is linked with a linguistic ability to evoke or reinforce these dichotomous representations in discourse in accordance with people’s social goals. Inspired by Chilton’s Discourse Space Theory (Chilton, 2004), the proximization approach, also called the Legitimization-Proximization Model (LPM), was initially developed in Cap’s analysis of interventionist discourse concerning US rhetoric during the Iraqi war (Cap, 2006). Proximization is defined by Cap (2008, 2015) as a construal operation meant to evoke closeness of the external threat to solicit legitimization of preventive measures. It is a more comprehensive cognitive linguistic approach to consider the ideological load of linguistic structures in terms of the conceptual processes they invoke and focuses mainly on categorization, spatial representation, and deixis, which bring into effect a range of ideological discursive strategies (Cap, 2013, 2017). Cap (2021) argues that the LPM subsumes a dynamic conception of discourse space, involving not only the opposition between the Self and the Other but also the discursively constructed movement of the Other toward the Self. This reveals a linguistic focus on the lexical and grammatical deictic choices that speakers make to index the existing socio-political and ideological distinctions and to demonstrate how the Other is constructed as erasing these distinctions by forcibly colonizing the in-group’s space. In that sense, this model can be described as a theory of coercion and threat construction (Cap, 2021).

**FIGURE 1:**
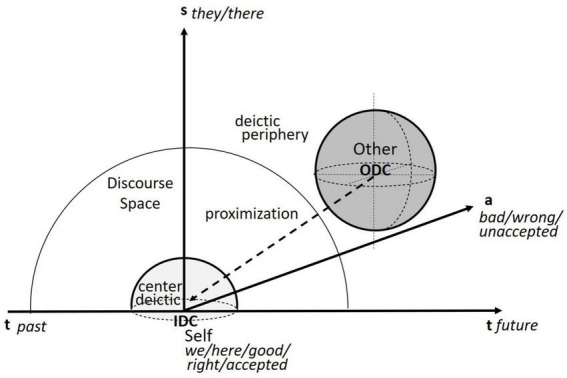
Representations of discourse space based on Cap (2017:5–6).

When analyzing the discourse of threat, the proximization framework works with two antagonistically constructed discourse spaces, one related to the speaker in the deictic center (Inside-Deictic-Center, IDC) and the other located at the periphery of this discourse space (Outside-Deictic-Center, ODC) (Cap, 2017). As is shown in Figure 1, the immediate threat is construed in a way that the antagonistic ODC is gradually approaching spatially (vertical axis), temporally (horizontal axis), and axiologically (stacked axis), and threatening the IDC or even taking it over (Cap, 2017). Specifically, the spatial markers, such as *we and they* or *here and there*, etc., located on the spatial axis are the core of the linguistic representation, which represents binary oppositions extending into the other two dimensions. The temporal axis in terms of *past and future* construes the threat as not only imminent but also momentous, historic and thus needing immediate response and unique preventive measures (Cap, 2014). The axiological axis concerns beliefs and values of the deictic center Self (*good, right, accepted*, etc.) in contrast to the discourse periphery Other (*bad, wrong, unaccepted*, etc.). Crucially in Cap’s LPM model, the axiological proximization framework categorizes ideological discourse choices in terms of distinct lexico-grammatical items, phrases, and discursive sequences, which enables qualitative and quantitative analyses of the core language items and formulae that make up the discourse and the ideological-material transformation (Cap, 2010, 2021).

### 2.3. An analytical framework for security discourse

**FIGURE 2:**
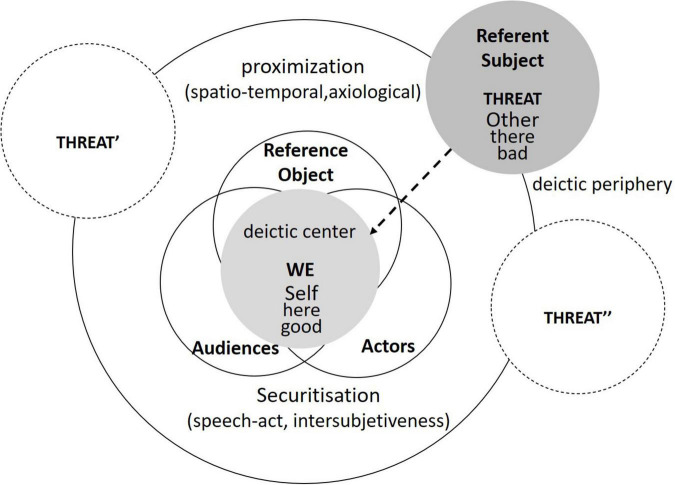
A discourse-based analytic framework.

Drawing on the similarity of discursively constructed threats to legitimate an urgent exceptional action, this article uses the proximization approach to demonstrate how an issue is securitized discursively and socio-politically. As is shown in Figure 2, in spite of employing the analyzing units of securitization theory, i.e., the securitizing actor, the referent subject, the referent object, and the audience, this framework focuses more on the intersubjective meaning-making process of reaching an agreement on the security issue, conferring an intersubjective consent status to the threat. For a successful securitization, the intersubjective cognition evoked into a WE perspective against the referent subject should be achieved by the discursive construction of the issue as a common existential threat, which, meanwhile, suggests a proximization process through which discursive strategies are applied spatio-temporally and axiologically to legitimate exceptional actions.

Rather than applying the state-of-art account of the securitization theory by locating the speech act, the securitizing actor, and the audience within a constative-performative continuum, this study uses a process-oriented account to reveal how it mobilizes securitization iteratively through a dynamic securitization trinity. First, the securitizing actor is decentered and the audience is further examined with their ideational and material investment in the securitizing move. A securitizing actor is more concerned with the dual roles, i.e., a speaker and a listener, who produces discourse-in-process with a mixture of the securitizing moves in the course of securitization, while the audience moves from a proscriptive subject interpellated by the securitizing actor to an agent whose everyday life is integral to securitization. In other words, both the securitizing actor and the audience are enacted through the securitizing move and are subjects-in-process. As indicated above, the audience plays a constitutive role in the securitizing move both before and after the securitized utterance *via* their investment. Ideationally, it is the audience’s recognition of the subject positions and discursive apparatuses that enable the securitized utterance to circulate. Materially, it is their implicit, embodied assumptions that are incarnated in audience behavior that make securitization possible. For investigating the speech act and linguistic practices, this study aims to provide a cognitive approach to demonstrate how the securitization is realized in form of the intersubjectiveness, which is linguistically and cognitively constructed through legitimizing the referent subject as a threat to the Self inside the deictic center. It is worth noting that this framework is providing a static set of cognitive discourse analytical tools to understand the securitizing move, but securitization is a dynamic process-oriented trinity along with the historical and socio-political practices of the issue.

## 3. Case study

### 3.1. Context of the situation

Along with the enforcement of national security strategies, the US security agenda covers a much wider area of national and international issues, extending from the conventional political sphere to all other unconventional spheres, e.g., the environment, economy, society, education, etc. Recently, the US uses security-related discourses and incites possible security shocks from foreign-based educational and cultural exchange programs. The Confucius Institutes, declaring to strengthen Chinese educational and cultural cooperation with countries around the world, has recently been drawn to a closure in the United States for the sake of securing the country. As we can see, more and more opinions in the public discourses now have been using national security as an excuse to scrutinize the role of the CIs, which have a great incitation on civilians as well as a bottom-up influence on government policy.

Table 1 displays the timeline of key events concerning Confucius Institutes in the United States. Based on the key events, the evolution of Confucius Institutes in the US can be summarized into three periods, namely, the positive reception from 2004 to 2013, the cognitive transformation from 2014 to 2020, and the post-closure period from 2021 to the present.

**TABLE 1:** Timeline of the key events concerning Confucius Institutes in the US.

Period	Timeline of the events (year)	Actors
Positive reception (2004—2013)	First CI in University of Maryland (2004-11)	Maryland University
	The CI teachers’ visas problem (2012-05)	Department of State
	81 CIs established in US (2013-12)	American Universities
Cognitive transformation (2014—2020)	CI closure in University of Chicago (2014-09)	Chicago University
	CI closure in Penn State University (2014-10)	Penn State University
	Report (2017-04)	NAS
	Hearing on “Worldwide Threats” (2018-02)	FBI Director
	The National Defense Authorization Act (2018-08)	Congress
	Report (2019-02)	GAO
	Report (2019-02)	Committee on HSGA
	CI US Centre as a foreign mission (2020-08)	Department of State
	Report (2020-08)	NAS
Post-closure (2021 to present)	More groups against CIs, e.g., AI (2021-present)	The Athenai Institute
	85 CIs closed or to be closed (2021-07)	American Universities
	Report (2022-06)	NAS

#### 3.1.1. The positive reception

In its initial establishment years from 2004 to 2013, the program won great popularity in the local American Universities since the first Confucius Institute hosted by the University of Maryland launched in 2004. On 17 May 2012, the Department of State requested teachers of Confucius Classrooms holding J-1 visas must return to China before 30 June to reapply for a suitable visa, and all *CIs* were required to obtain US academic accreditation (Fischer, 2012). Although the requirement was later withdrawn from the swamp of objections, the attitudes of the US toward the *CIs* have gradually changed. In 2013, there were 81 Confucius Institutes around the United States.

#### 3.1.2. The cognitive transformation

The year 2014 witnessed the first closure of the Confucius Institute, established in 2010 at the University of Chicago, in the United States. The closure marked a turn in Confucius Institutes’ development in the US and symbolized the start of the cognitive transformation period. During the years from 2014 to 2017, the negative attitudes toward CIs increased gradually from an “educational institution” to a “political agency,” which contributed to the beginning transformation of the perceptions of CIs in the US (Lien and Tang, 2021). As an influence of professional opinions representing American education groups, NAS published its first report entitled “Outsourced to China: Confucius Institutes and Soft Power in American Higher Education” (National Association of Scholars, 2017), citing CIs as interfering with academic freedom and calling for the closure of CIs. In February 2018, the Federal Bureau of Investigation (FBI) Director Christopher Wray said at the hearing on “Worldwide Threats” that the FBI was trying to view the China threat as not just a whole-of-government threat but a whole-of-society threat. As a bipartisan consensus in the policy toward China, the US passed the National Defense Authorization Act (NDAA) in August 2018 to force the schools to pick between CIs and the Chinese Language Flagship Programme funded by the Department of Defense, symbolizing a start to use legislative measures against CIs. On 28 February 2019, a report named “China’s Impact on the U.S. Education System” was released by Committee on Homeland Security and Governmental Affairs. Following this in 2020, Senator James Lankford submitted the Transparency for Confucius Institutes Act to secure American universities from political propaganda through CIs, and later the US Department of State designated the Confucius Institute US Centre as a foreign mission of the People’s Republic of China. Nevertheless, in 2020, NAS published another report entitled “Corrupting the College Board: Confucius Institutes and K-12 Education,” further citing *CIs* as threatening the US education system (National Association of Scholars, 2020).

#### 3.1.3. The post-closure period

Confucius Institutes in the US evolves a rapid increase in closure and more education groups join in pushing this action, e.g., a newly established organization called the Athenai Institute declaring it to be their first work. By July 2021, an estimated number of 85 Confucius Institutes are closed or to be closed in American universities. In June 2022, NAS published its third report titled “After Confucius Institutes: China’s Enduring Influence on American Higher Education,” documenting what happens when Confucius Institutes close (National Association of Scholars, 2022).

According to the above timeline of the CI-related socio-political events, we generalize the following units according to the dynamic process-oriented securitization framework proposed in the previous section:

• Securitizing actors: There are political agents (senators, US governmental institutions, etc.) and public agents (American university administrators, academia elites or groups, public media, etc.), who are actors of securitizing moves in different periods.
• Securitizing audiences: In response to the securitizing actors in different periods, the audiences accordingly are political and public agents due to their addressee’s position in the discourse context.
• Referent subject: Confucius Institutes as “existential threat.”
• Referent object: This largely depends on the degree to which the securitizer’s intention to identify how serious the threat is. In our concerned case, it is a threat originally to US academic freedom, then to US education, and then to US national security.

In summary, the agents involved in the diachronic evolution of Confucius Institutes in US discourse play a dynamic and interactive role during different periods. One agent can be passive in being an audience and active in delivering a move, having a dual role in the interactive meaning-making process. What arouses our strong interest is how the cognitive transformation has been successfully worked out to be an intersubjective commonness and discursively constructed in the discourse, and especially what exactly happened in the agents’ cognitive process of making an agreement toward Confucius Institutes from the transformation of an academic issue to a security one.

### 3.2. Data collection and the corpus

**FIGURE 3:**
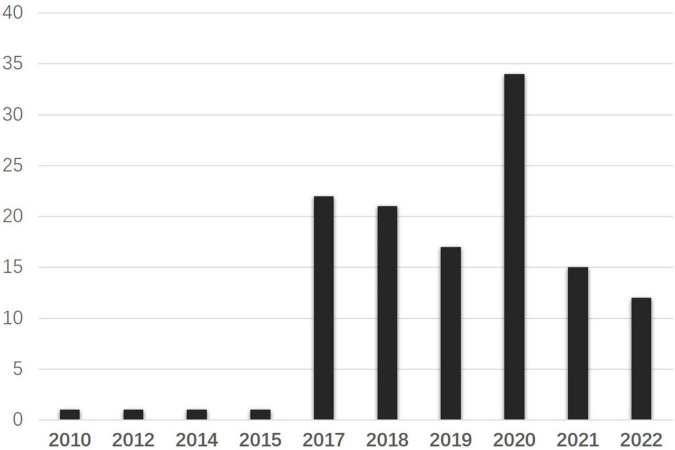
Frequencies of texts pertaining to Confucius Institutes over time.

The National Association of Scholars, one of the American academic groups that keep active and constant concern on Confucius Institutes, claims its mission to defend academic freedom, investigate issues affecting academic freedom, and educate the public to protect academic freedom. It will be interesting to know what they, representing US academic agents and their interests, speak to conceptualize CIs and appeal to act toward CIs diachronically. Therefore, this study collected all the texts concerning Confucius Institutes on NAS’s official websites. By the date of 30 September 2022, there were 125 search results by the search word “Confucius Institutes” in Figure 3. In general, NAS has an increasing concern about Confucius Institutes along with the climbing number of CIs*-*related texts on the official website. In 2014, an article titled “The New Problem of Higher Education: The Foreign-based Institute” was published online, questioning the Confucius Institutes and their influence in the US, which echoed the US concerns and reflections on Confucius Institutes. Then, there was a silent period till 2017, as an in-depth report to investigate whether Confucius Institutes affected US academic freedom, NAS published their findings in form of an academic report titled “Outsourced to China: Confucius Institutes and Soft Power in American Higher Education” worldwide. Since then, the online discourse went on the rise with a summit of 35 texts published in 2020, and this represents NAS’s active involvement in pushing the securitizing move on Confucius Institutes.

During the cognitive transformation period from 2014 to 2020, NAS delivered varieties of discourses against Confucius Institutes, pushing the public, university administration, and state administration to close CIs. Focusing on this specific period and eliminating the unrelated texts with a later screening of its content, the corpus finally was made up of 68 texts consisting of 156,268 words (Table 2). Since the *National Defense Authorization Act for Fiscal Year 2019* was launched in 2018, symbolizing a bipartisan consensus in the use of state legislation against CIs, we define the year 2018 as a critical turning point for the federal top-down control over Confucius Institutes, which exerts a direct influence on the further cognitive transformation of CIs in the United States, and therefore is the boundary between Sub-corpus I (2014–2017) and Sub-corpus II (2018–2020). All the texts in the corpus were converted from their original, varied formats to a uniform text format amenable to analysis. For a convenient data read, texts are coded with a file name format of “year-text + serial number-author,” for example, “2020-Rept01-NAS” refers to the first report produced by NAS in 2020.

**TABLE 2:** The corpus: CI-related texts on NAS’s official website from 2014 to 2020.

Corpus	Years	Texts	Size	Tokens	Types
Sub-corpus I	2014–2017	16	100,065	81,688	6,286
Sub-corpus II	2018–2020	52	56,203	48,130	5,284
Total	2014–2020	68	156,268	129,818	8,580

With the purpose of comparing the two sub-corpora against each other, the corpus-based technique of keyword analysis was employed, using PowerConc 1.0 developed by the Corpus Research Group of BFSU. As is agreed in previous corpus-supported discourse studies (Leech and Fallon, 1992; Fairclough, 2000; McEnery, 2005; Hunter and Smith, 2012), to compare lexical frequency information across two corpora can expose differences between them, and therefore the cross-comparison of diachronically divided sub-corpora can suggest historical differences. The keywords comparison investigated the frequencies of all the words in the 2014–2017 and the 2018–2020 sub-sections of the corpus with each other in order to ascertain which words occurred statistically more often. Such keywords are supposed to elucidate the most salient ways in which Confucius Institutes are discursively constructed in the two consecutive historical phases. Although the lists of words reveal very little on their own without context, the keyword analysis was supplemented with concordance analyses. Where it is needed, examples are taken from the original text that would be illustrative of contextual uses of particular words.

### 3.3. Results and discussion

What can be drawn in common is that the two sub-corpora resemble in the frequent use of academic terms like *interview, report, found, study*, the third pronoun *he, it*, *she*, the third person, and the direct quotes, signifying the genres and styles used for the professionalism and objectivity in a particular academic discourse (Hyland, 2009). Despite those common language features, the two sub-corpora remain significantly different in the keywords. A statistical keywords comparison was carried out by comparing wordlists derived from Sub-corpus I and Sub-corpus II. Using the log-likelihood statistical test, each word is investigated on the strength of the difference. The 100 strongest lexical keywords for Sub-corpus I and Sub-corpus II can be found in Supplementary Appendixes 1, 2.

Having obtained the keyword lists, the next task is to examine as many of the words as possible in order to unveil the major differences in the representation of Confucius Institutes between the two conservative phases. Following Baker, 2010a,b, the study proceeds based on that the top 100 words identified as statistically key in each sub-corpus should be investigated as candidates for significance, using further quantitative checks and manual, context-sensitive qualitative assessment to support claims of “salience” (Baker, 2006, p. 125). Thus, we first checked the senses and roles displayed by the keywords when checked in context *via* concordance. Next, we looked at statistical data relating to the collocation of keywords, or their tendency to appear in combination or the company of other words. A list of collocations using the default horizon of five words to the left and right of each term was also considered. Then, we considered the clusters of words that regularly formed around the keywords within each sub-corpus. Finally, the linguistic data were grouped together under emergent themes observed in the data.

#### 3.3.1. Discourse genre from academic narratives to security narratives

**FIGURE 4:**
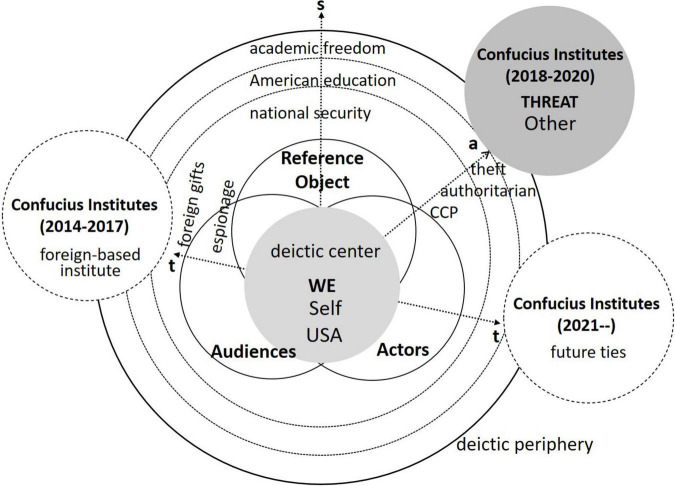
The security discourse of Confucius Institutes.

Based on the corpus statistics above, the securitizing case of Confucius Institutes would be further discussed within the cognitive critical discourse analytical framework proposed previously. As is put as a focus in the framework, securitization is a dynamic process-oriented interaction among the securitizing agents, in which the cognitive “Self-Other” dichotomy would be intersubjectively activated and enhanced. Rather than specifying the securitizing actor of a particular move, we consider the securitization of Confucius Institutes as a dynamic co-operated process under the complex socio-political context. In line with the socio-political events happening diachronically, Figure 4 shows the diachronic change of Confucius Institutes’ identity in different phases and a synchronic process of constructing Confucius Institutes as an existential threat outside the deictic center approaching close to the USA, the Inside Deictic Center, in the discourse space. The closure of Confucius Institutes means the urgent exceptional actions called upon against the threat, then what the involved agents are cognitively undergoing would in turn indicate the meaning-making process of security construction.

The collected texts in the corpus are produced by NAS, which display the ways American elites write about Confucius Institutes. Previously in the one-word N-gram lists of the two sub-corpora, the quotation marks hit the top frequencies. It is obvious that the discourses present a constant style of quoting to convince the reader of the credibility of their stories about Confucius Institutes. When further exploring the data, the study finds that the *“quotation marks”* do not present in the keywords list in Sub-corpus II. In contrast, there is more than one hit per one hundred words in Sub-corpus I (Freq. = 0.0146, Ref-Freq. = 0.0095, Log-likelihood = 62.3234), which conversely indicates less use of direct quotes in Sub-corpus II. Then with more careful analysis of the concordance, the results turn out that the indirect quotes from political agents as senators and government officials are dominantly used when it comes to the national security threat, and therefore mixes a style of political narratives in Confucius Institute’s discourses. As is acknowledged in excerpt (1), a list of political authorities is quoted here to show the political consensus in taking urgent political actions toward Confucius Institutes, and the intersubjectiveness in securitization has also been achieved by expressing NAS’s positive position toward them.

(1) Meanwhile, the Senate has passed a new, *bipartisan bill* on its way to the House: *the CONFUCIUS Act* (Concerns Over Nations Funding University Campus Institutes in the United States Act). The bill was introduced by *Senator John Kennedy (R-LA) and is cosponsored by Senators Doug Jones (D-AL), Chuck Grassley (R-IA), and Marsha Blackburn (R-TN)*. In this week’s featured statement, NAS breaks down the key provisions of the bill, *applauds these Senators for their fine work*, and proposes amendments to the bill. (Source Text: 2020-Art06-JD).

By fostering a cognitive-affective differentiation and identification, “Self-Other” security narratives evoke collective sentiments of both aggression and empathy. The line between ingroup and outgroup is, therefore, intentionally and unambiguously drawn. In other words, the ways in which Confucius’s Institutes are constructed work to create dichotomous characteristics of the opposing sides and are not to tell us what to think but to inform us what to think about because they communicate a code that information contained is interpreted. What matters most in terms of their broader socio-political context is that the protagonist-antagonist frame inculcates a preference for taking urgent and exceptional political actions for one’s own sake. Excerpt (2) taken from NAS’s report in 2020 displays how the political narratives work out when a particular matter is regarded as a security threat. Confucius Institutes are defined as an outside group referring to China, a competitive geographical adversary, which mixes the NAS’s academic discourses with political narratives of dividing the world into a “us” and “enmity” dichotomy.

(2) Federal Bureau of Investigation Director Chris Wray has warned that Confucius Institutes are part of *China’s “whole-of-society” threat to American freedoms*. This year, Secretary of State Mike Pompeo designated *the Confucius Institute U.S. Center as a “foreign mission” of the People’s Republic of China*, calling it “an entity advancing *Beijing’s global propaganda* and malign influence campaign on *U.S. campuses and K-12 classrooms*. Last February, the Senate Permanent Subcommittee on Investigations concluded in a 96-page report that Confucius Institutes operate as *“part of China’s broader, long-term strategy” to develop “soft power” and “export China’s censorship” to college campuses*. (Source Text: 2020-Rept01-NAS).

#### 3.3.2. The cognitive transformation from “academic malware” to “security threat”

In the wordlist of Sub-corpus I, keywords including *Course, interview, director, Professor, Studies, class, Hiring, topics, Criteria, teachers, and disputes* sketch the reflection of Confucius Institutes as a foreign-based institute, concerning its negative influence on academic freedom from 2014 to 2017. As is quoted directly in National Association of Scholars’s (2017) report, Marshall Sahlins, a professor emeritus of Anthropology at the University of Chicago, collects dozens of instances of Confucius Institutes’ interference, censorship, or pressure to self-censor and identifies it as the academic malware in his book Sahlins (2014). In line with this, texts in Sub-corpus I indicate the disputes over Confucius Institutes in American academia, even the referent objects in the threat discourses of Confucius Institutes are lingering on academic freedom and education. Relatively speaking, there are many more direct references in the sub-corpus II to associate Confucius Institutes with national security from 2018 to 2020. Sub-corpus II keywords, including *Act, espionage, transparency, illegal, Security, defense, Threat, investigations, and legislation*, construct a security discourse, in which Confucius Institutes as a national threat are established and exceptional political actions are called on to be taken. In addition, the keywords *government*, *colleges, federal, universities, Scholars, public, Senate, Congress, and Department* together identify the agents involved in the agreement of securitizing Confucius Institutes. Such keywords significantly mark the diachronic cognitive difference in the representation of Confucius Institutes in the discourses.

It is worth noting that the cognitive transformation of CIs’ identity is not a point of time event and it evolves over a matter of years. By comparison of the sub-corpora of two different phases, the results turn out that the years from 2018 to 2020 witnessed the cognitive transformation of Confucius Institutes to be a security issue in the United States. That is to say, the discourses in this phase will show discursive strategies for building Confucius Institutes into a threat to American national security. According to the keyword concordance during this period, Confucius Institutes are narrated to be an existential threat in many ways to US national security. Critically in the keyword list, *gifts* (Log-likelihood = 152.61) as a starting point in constructing the security discourse. With its most frequent concordance word *foreign* (Freq. = 41.03%), it refers to a foreign source of funds from foreign individuals, organizations, and governments.

(3) The problem lies not only in the content of the law: enforcement of the meager regulations currently in place has been scant at best. For example, *nearly 70% of colleges receiving Chinese-government funding for Confucius Institutes never reported their gifts to the Department of Education (ED)*. The same two universities listed above also received over $250 million undisclosed dollars from the nation of Qatar. Why? No one knows. We see that even gifts ten times the size of the established threshold can be kept secret by schools that simply neglect to report them; *anyone who values national security or the financial self-reliance of higher education should find this wholly unacceptable*. Once the law is changed, it needs to be enforced strictly in order for foreign giving to be truly transparent. (Source Text: 2019-Art01-JD).
(4) Undisclosed foreign funding in American higher education is one of the most pervasive threats to the academy and national security. Every day, *geopolitical adversaries pour untold amounts of secret money into U.S. colleges and universities to buy influence and exert soft power from within*. While *China is likely the most flagrant offender in this area*, it is far from alone. Other nations–Qatar, Saudi Arabia, and Iran–*play the dark money game* with American colleges and universities. (Source Text: 2020-Art04-JD).

As is described in (3) and (4), the foreign source of the fund was questioned in terms of *transparency* (Log-likelihood = 48.17), in particular, dark money brought by Confucius Institutes is the most flagrant one to put the American universities, education, and the whole country in a danger. While traced back to the National Association of Scholars’s (2017) report, it proposes the question of how much influence has been exerted by Confucius Institutes since American colleges and universities had set up Confucius Institutes funded and largely staffed by the Chinese governments, which drew a conclusion that American higher education was outsourced to China and influenced by its soft power. Comparatively, in the discourses of 2018 to 2020, this situation was further aggravated by the dismal level of transparency within Confucius Institutes and the corrupted American higher education system. Subsequently, *government* (Log-likelihood = 125.10) hit a high keyness next to *gifts* in the keywords list, showing a more inclination that Confucius Institutes were nothing but agencies of the Chinese government, a geopolitical adversary of the USA, which seemed to be the root of non-transparency of fund and management in Confucius Institutes. Therefore, Confucius Institutes were cognitively constructed as an equivalent of the Chinese government.

(5) The National Association of Scholars has called for colleges and universities to close their Confucius Institutes, *citing extensive evidence that Confucius Institutes undermine academic freedom, present students with a one-sided view of China, and entangle colleges and universities in a web of financial relationships that leave them dependent on China*. Our report, Outsourced to China, remains a comprehensive look at the way the Chinese government works to coopt American colleges and universities. We also note that the FBI and multiple members of Congress are also concerned that in addition to undermining academic freedom, *Confucius Institutes may jeopardize national security*. (Source Text: 2018-Art08-NAS).

Meanwhile, *Security* (Log-likelihood = 42.09) in the Sub-corpus II keyword list upgrades the level of potential insecure fields Confucius Institutes would interfere with and influence. By searching for its concordance words, *national* security ranked the top one in the Collocation and Colligation list with a Log-likelihood of 237.63. Representing the official statement of NAS, (5) is an excerpt taken from the text published in 2018. Although used as a hedge word presenting information as an opinion rather than absolute fact, the stance-taking marker *may* was used to guide the public to the proposition that Confucius Institutes possibly have harmed American national security. Consequently, the version of “security threat” starts to take shape in discourse.

#### 3.3.3. Legitimization-proximization from a “bargain” to an “espionage”

From the constructive perspective, the intersubjectiveness in securitization is legitimized by evoking the process of conceptualizing the outside threat in terms of spatial, temporal, and axiological proximization.

Temporally, the keywords of the two sub-corpora present a diachronic change in the identity from a foreign-based institute in 2014–2017 to a security threat in 2018–2020. The first Confucius Institute was established in 2004 at the University of Maryland, which started the popularity of winning a host of Confucius Institute in American universities and colleges, exemplifying a wide cooperation in cultural exchange and language education between the two countries. By the end of 2013, there was an estimation of 81 Confucius Institutes open on American university campuses. A “bargain” as it is in (6), the host universities make an agreement with CIs program in co-operating with Confucius Institutes and gain an amount of money for the cost of running the institutes. With an operating fund, the CIs program is regarded as one of the foreign-based programs in American universities that offer a solution to the financial problems of US higher education. American education groups represented by the American Association of University Professors (AAUP) and NAS express legitimate concerns about the relationship between Confucius Institutes and American universities, and those concerns are apparently within their profession or expertise at the moment.

(6) The Confucius Institute is *a bargain* between the Hanban, a Chinese government agency, and university administrators. *Such institutes allow the Chinese Government regular access to western culture. In return, and at no monetary cost, university administrators gain favorable publicity and an instrument to recruit students*. In exploring this particular relationship, NAS will be joined by an array of other higher education groups such as the AAUP who have legitimate concerns. (Source Text: 2014-Art01-JAS).

Data from later texts in the corpus reveal that concerns of American academic elites come up with the securitizing move delivered by the political agents toward Confucius Institutes. The *National Defense Authorization Act for Fiscal Year 2019* is launched by the House and Senate in 2018, in which a key amendment is put forward to prohibit funding under the act to any Confucius Institutes and restrict funding to any college or university that has a Confucius Institute. These top-down political discourses draw the public to a unified cognition that the CIs program is an existential China threat to national security more than an academic issue.

Spatially, Table 3 illustrates the statistical results of the frequencies of grammatical and lexical items concerning spatial proximization. The core components of IDCs are *American*, *Universities*, *colleges*, *America*, *we*, etc. (Total Freq.% = 3.34). At the other end of the event stage, ODCs include the *Confucius Institutes*, *Confucius Classrooms*, the *Chinese government*, the *Chinese Communist Party (CCP)*, *they*, etc. (Total Freq.% = 4.98). A relation of shared identity is established between some of these elements. *Confucius Institutes*, the *Chinese government*, and the *Chinese Communist Party* are put on common ground through the sheer proximity of their lexical occurrence in the texts of Sub-corpus II. The relative distance between IDCs and ODCs is shrinking as a result of the actions indicated by lexical items in Categories 3 and 4 in Table 3. As the initiator, Confucius Institutes have been taking illegal actions, such as *interfering*, *infiltrating*, *stealing*, *corrupting*, and *spying*, thus *influencing* American universities and colleges, *compromising* the integrity of American higher education, *undermining* American academic and intellectual freedom, and *jeopardizing* American national security. Lexical items in Category 5 show the existing results of ODCs’ influence over IDCs: *foreign gifts* in American universities and colleges, *soft power* exerting on American education, *propaganda* over Americans, and other *Chinese (China) influence*.

**TABLE 3:** The key lemmas of the spatial framework of the Confucius Institute rhetoric, 2018–2020.

Category	Key lemma/Concordance	Frequency (%)
1	American(s), University(-ies), college(s), America, we	3.34
2	Confucius Institute(s), Confucius Classroom(s), CI, Chinese government, Chinese Communist Party (CCP), China, they	4.98
3	Interfere, infiltrate, steal, corrupt, blur, spy	0.23
4	Influence, compromise, jeopardize, undermine	0.18
5	freedom/security threat, danger	0.34
6	foreign gifts, soft power, propaganda, Chinese (China) influence	0.74
Total		9.82

(7) Our report found that Confucius Institutes are Chinese government-sponsored centers located at colleges and universities around the world. (There are currently more than 80 in the United States.) *Confucius Institutes present a heavily edited version of the Chinese Communist Party’s authoritarian rule and educate a generation of American students to know little more about China than the regime’s official history*. (Source Text: 2020-Art07-RP).
(8) Alabama is poised to become the first state to *take up legislation* banning public colleges and universities from hosting Confucius Institutes, the Chinese government-sponsored campus centers *that propagandize for Beijing and serve as outposts of Communist Party espionage.* (Source Text: 2020-Art01-RP).

As has already been indicated in the previous section, the spatial axis involves entities conceptualized in different and variable degrees of physical and geopolitical distance from the discourse addressee located within the deictic center, while the perception by the addressee in terms of the current positive and negative value characteristics would be further conceptualized in the axiological axis. There are profound and complex historical origins between the United States and China, yet the difference lies essentially in the two kinds of ideology. Since Confucius Institutes represent China, the ideological concepts of China, especially the Chinese Communist Party, are approached as alien ideologies. The linguistic enactment of values antithetical to those of the deictic center would naturally be rejected. The drastic imagery of “authoritarian” in (7) would activate the audience’s unlimited prototype about the uncivilized and fear of the alien ideology extremely opposite to American democracy. Furthermore, “espionage,” “theft,” and “spy” point directly to national security so that Cold War mentality would cognitively be activated in the audience. Example (8) shows us the state legislation taken up by the State of Alabama, and the legitimation of taking the political action is achieved by the proximization of being threatened by an alien ideology and its espionage.

## 4. Conclusion

Integrating securitization theory with the cognitive approach of CDS, the study finds that the American academia deliver a bottom-up securitizing move by constructing education security discourse on Confucius Institutes in the initial process, yet later the whole-of-society security narratives interacted with a top-down securitizing move from the political agents have been adopted. As is indicated by the corpus statistics, NAS’s discourses of Confucius Institutes are discursively constructed by following the “Self-Other” dichotomy security narratives, in which CIs are cognitively transformed from an academic issue to a national security issue and legitimized through proximization in the spatial, temporal, and axiological dimensions. By focusing on a case study, this article aims to provide a better understanding of security discourses in two ways. First, how context constitutes the discourses. The context of discourse production and interpretation, especially the social, political, and ideological factors, impacts the American agents’ perceptions and also their later discourses of Confucius Institutes diachronically. Meanwhile, how the discourses constitute the society. Confucius Institutes are pushed to closure under the pressure of various political actions legitimized by drawing language devices in security discourses. The study displays that applying proximization as an explanatory approach to the securitization framework could work well for explaining how exactly those language devices work to realize the cognitive transformation in the agents and legitimizing the securitization toward the referent subject.

In discourse studies, texts are the research objects, which are significant in qualitative and quantitative research studies. The sampling and collection of texts will be critical to the reliability and validity of the results, while discourses are dynamically formed under the changing socio-political environment. Taking CI discourses for an instance, the securitizing actors and moves at a particular time vary a great deal diachronically. Therefore, the identification of securitization as a dynamic intersubjective meaning-making process will help to understand how the securitizing agents are cognitively and affectively involved. To better explain this, the study reviews the timeline of Confucius Institutes’ evolution in the US and concentrates on the particular period of time in which the cognitive transformation takes place, and this transformation will be discursively indicated in the discourses generated by different securitizing actors who may be the political actors, media actors, academic actors, and even later the audience themselves. In addition, the fact that the method to analyze discourses has not been agreed upon yet, semantic construal across texts is inevitably personalized and somehow subjective. The study attempts to interpret the texts by a data-driven corpus method instead of subjective projection. However, to keep an objective and unbiased analysis needs a corpus with a bigger size and more varieties of data, later studies could be designed and explored a lot in this aspect.

## Data availability statement

The datasets presented in this study can be found in online repositories. The names of the repository/repositories and accession number(s) can be found in the article/Supplementary material.

## Author contributions

CH designed and conducted the study, completed the statistical analysis, and wrote the manuscript.
