# Supplementary material for: A corpus-based study on the cognitive construction of security in discourse
Source: Front Psychol. 2023 Jan 6;13:1069896. doi: 10.3389/fpsyg.2022.1069896 (PMC9853886; doi:10.3389/fpsyg.2022.1069896)
Supplement: Supplementary file 1 [file Table_1.DOCX]

Supplementary Material

# Supplementary Data

Appendix 1. 100 strongest Sub-corpus I (2014-2017) keywords

| **N** | **Key word** | **Sub-corpus I** | | **Sub-corpus II** | | **Log-Likelihood** |
| --- | --- | --- | --- | --- | --- | --- |
|  |  | Freq. | % | Freq. | % |  |
| 1 | University | 1511 | 0.0185 | 285 | 0.0059 | 393.9957 |
| 2 | CI | 427 | 0.0052 | 4 | 0.0001 | 358.1318 |
| 3 | said | 341 | 0.0042 | 25 | 0.0005 | 183.0918 |
| 4 | Institute | 820 | 0.0100 | 191 | 0.0040 | 158.7481 |
| 5 | Agreement | 239 | 0.0029 | 12 | 0.0002 | 148.8457 |
| 6 | Course | 251 | 0.0031 | 16 | 0.0003 | 143.2004 |
| 7 | interview | 162 | 0.0020 | 1 | 0.0000 | 139.8884 |
| 8 | Hanban | 684 | 0.0084 | 169 | 0.0035 | 119.8351 |
| 9 | director | 337 | 0.0041 | 48 | 0.0010 | 117.8423 |
| 10 | Professor | 243 | 0.0030 | 27 | 0.0006 | 103.1635 |
| 11 | Article | 161 | 0.0020 | 10 | 0.0002 | 92.8184 |
| 12 | Jersey | 102 | 0.0012 | 2 | 0.0000 | 78.7008 |
| 13 | York | 119 | 0.0015 | 6 | 0.0001 | 74.0087 |
| 14 | I | 230 | 0.0028 | 36 | 0.0007 | 73.6343 |
| 15 | Asian | 96 | 0.0012 | 2 | 0.0000 | 73.3822 |
| 16 | Peterson | 210 | 0.0026 | 30 | 0.0006 | 73.239 |
| 17 | directors | 122 | 0.0015 | 7 | 0.0001 | 72.5107 |
| 18 | New | 268 | 0.0033 | 50 | 0.0010 | 70.8187 |
| 19 | Courses | 173 | 0.0021 | 22 | 0.0005 | 66.5093 |
| 20 | Studies | 125 | 0.0015 | 10 | 0.0002 | 64.3571 |
| 21 | class | 81 | 0.0010 | 2 | 0.0000 | 60.1574 |
| 22 | Tan | 77 | 0.0009 | 2 | 0.0000 | 56.6516 |
| 23 | Arts | 69 | 0.0008 | 1 | 0.0000 | 55.4271 |
| 24 | Hiring | 128 | 0.0016 | 14 | 0.0003 | 54.9268 |
| 25 | City | 67 | 0.0008 | 1 | 0.0000 | 53.6325 |
| 26 | she | 131 | 0.0016 | 17 | 0.0003 | 49.5572 |
| 27 | Description | 74 | 0.0009 | 3 | 0.0001 | 49.1581 |
| 28 | he | 282 | 0.0035 | 72 | 0.0015 | 46.5538 |
| 29 | Don | 75 | 0.0009 | 4 | 0.0001 | 45.7626 |
| 30 | topics | 76 | 0.0009 | 5 | 0.0001 | 42.7978 |
| 31 | Classes | 99 | 0.0012 | 11 | 0.0002 | 42.0296 |
| 32 | Chen | 53 | 0.0006 | 1 | 0.0000 | 41.1271 |
| 33 | Criteria | 53 | 0.0006 | 1 | 0.0000 | 41.1271 |
| 34 | Gong | 60 | 0.0007 | 3 | 0.0001 | 37.4186 |
| 35 | Falun | 59 | 0.0007 | 3 | 0.0001 | 36.5905 |
| 36 | Position | 53 | 0.0006 | 2 | 0.0000 | 35.8878 |
| 37 | Stephen | 47 | 0.0006 | 1 | 0.0000 | 35.8063 |
| 38 | some | 187 | 0.0023 | 44 | 0.0009 | 35.609 |
| 39 | very | 46 | 0.0006 | 1 | 0.0000 | 34.9224 |
| 40 | paid | 74 | 0.0009 | 7 | 0.0001 | 34.7923 |
| 41 | staff | 94 | 0.0012 | 14 | 0.0003 | 31.5616 |
| 42 | Constitution | 47 | 0.0006 | 2 | 0.0000 | 30.8003 |
| 43 | HTTP | 64 | 0.0008 | 6 | 0.0001 | 30.2485 |
| 44 | Chinese | 1268 | 0.0155 | 569 | 0.0118 | 30.074 |
| 45 | Teaching | 104 | 0.0013 | 18 | 0.0004 | 29.9768 |
| 46 | email | 39 | 0.0005 | 1 | 0.0000 | 28.7636 |
| 47 | her | 78 | 0.0010 | 11 | 0.0002 | 27.5153 |
| 48 | teachers | 256 | 0.0031 | 80 | 0.0017 | 27.0847 |
| 49 | not | 326 | 0.0040 | 112 | 0.0023 | 26.2597 |
| 50 | reputation | 50 | 0.0006 | 4 | 0.0001 | 25.7428 |
| 51 | Offered | 68 | 0.0008 | 9 | 0.0002 | 25.3159 |
| 52 | Economics | 45 | 0.0006 | 3 | 0.0001 | 25.1997 |
| 53 | books | 49 | 0.0006 | 4 | 0.0001 | 24.9718 |
| 54 | Capital | 38 | 0.0005 | 2 | 0.0000 | 23.2929 |
| 55 | role | 82 | 0.0010 | 15 | 0.0003 | 22.1855 |
| 56 | Professors | 123 | 0.0015 | 30 | 0.0006 | 22.0425 |
| 57 | Agreed | 41 | 0.0005 | 3 | 0.0001 | 22.0338 |
| 58 | Wang | 80 | 0.0010 | 15 | 0.0003 | 21.0122 |
| 59 | appointed | 29 | 0.0004 | 1 | 0.0000 | 20.0829 |
| 60 | BU | 29 | 0.0004 | 1 | 0.0000 | 20.0829 |
| 61 | visiting | 29 | 0.0004 | 1 | 0.0000 | 20.0829 |
| 62 | Tiananmen | 62 | 0.0008 | 10 | 0.0002 | 19.2611 |
| 63 | regular | 28 | 0.0003 | 1 | 0.0000 | 19.2254 |
| 64 | requests | 28 | 0.0003 | 1 | 0.0000 | 19.2254 |
| 65 | there | 104 | 0.0013 | 25 | 0.0005 | 19.108 |
| 66 | me | 48 | 0.0006 | 6 | 0.0001 | 18.7026 |
| 67 | Link | 27 | 0.0003 | 1 | 0.0000 | 18.3704 |
| 68 | faculty | 102 | 0.0012 | 25 | 0.0005 | 18.1243 |
| 69 | Headquarters | 60 | 0.0007 | 10 | 0.0002 | 18.0154 |
| 70 | Square | 63 | 0.0008 | 11 | 0.0002 | 17.9826 |
| 71 | study | 71 | 0.0009 | 14 | 0.0003 | 17.504 |
| 72 | able | 42 | 0.0005 | 5 | 0.0001 | 16.978 |
| 73 | Draft | 33 | 0.0004 | 3 | 0.0001 | 15.8741 |
| 74 | felt | 24 | 0.0003 | 1 | 0.0000 | 15.8221 |
| 75 | Beijing | 65 | 0.0008 | 13 | 0.0003 | 15.7298 |
| 76 | case | 56 | 0.0007 | 10 | 0.0002 | 15.5826 |
| 77 | Associate | 46 | 0.0006 | 7 | 0.0001 | 15.1348 |
| 78 | Dean | 32 | 0.0004 | 3 | 0.0001 | 15.1243 |
| 79 | Office | 61 | 0.0007 | 12 | 0.0002 | 15.0844 |
| 80 | personal | 23 | 0.0003 | 1 | 0.0000 | 14.979 |
| 81 | because | 85 | 0.0010 | 21 | 0.0004 | 14.8936 |
| 82 | Culture | 143 | 0.0018 | 45 | 0.0009 | 14.8542 |
| 83 | contract | 66 | 0.0008 | 14 | 0.0003 | 14.732 |
| 84 | pressures | 27 | 0.0003 | 2 | 0.0000 | 14.4278 |
| 85 | Willingness | 27 | 0.0003 | 2 | 0.0000 | 14.4278 |
| 86 | Xu | 31 | 0.0004 | 3 | 0.0001 | 14.3798 |
| 87 | avoid | 38 | 0.0005 | 5 | 0.0001 | 14.2152 |
| 88 | events | 38 | 0.0005 | 5 | 0.0001 | 14.2152 |
| 89 | did | 65 | 0.0008 | 14 | 0.0003 | 14.193 |
| 90 | Rachelle | 103 | 0.0013 | 29 | 0.0006 | 13.9715 |
| 91 | were | 110 | 0.0013 | 32 | 0.0007 | 13.8703 |
| 92 | partner | 47 | 0.0006 | 8 | 0.0002 | 13.7972 |
| 93 | told | 46 | 0.0006 | 8 | 0.0002 | 13.1883 |
| 94 | Cooperation | 33 | 0.0004 | 4 | 0.0001 | 13.1627 |
| 95 | speech | 42 | 0.0005 | 7 | 0.0001 | 12.6108 |
| 96 | Policies | 80 | 0.0010 | 21 | 0.0004 | 12.5296 |
| 97 | disputes | 20 | 0.0002 | 1 | 0.0000 | 12.4729 |
| 98 | examined | 20 | 0.0002 | 1 | 0.0000 | 12.4729 |
| 99 | side | 20 | 0.0002 | 1 | 0.0000 | 12.4729 |
| 100 | Languages | 24 | 0.0003 | 2 | 0.0000 | 12.1019 |

Appendix 2. 100 strongest Sub-corpus II (2018-2020) keywords

| **N** | **Key word** | **Sub-corpus II** | | **Sub-corpus I** | | **Log-Likelihood** |
| --- | --- | --- | --- | --- | --- | --- |
|  |  | Freq. | % | Freq. | % |  |
| 1 | colleges | 257 | 0.0053 | 45 | 0.0006 | 297.4222 |
| 2 | foreign | 251 | 0.0052 | 80 | 0.0010 | 206.1138 |
| 3 | gifts | 117 | 0.0024 | 15 | 0.0002 | 152.6086 |
| 4 | National | 200 | 0.0042 | 73 | 0.0009 | 147.4846 |
| 5 | Education | 260 | 0.0054 | 133 | 0.0016 | 136.1496 |
| 6 | government | 268 | 0.0056 | 150 | 0.0018 | 125.0991 |
| 7 | Act | 84 | 0.0017 | 10 | 0.0010 | 112.2483 |
| 8 | College | 213 | 0.0044 | 111 | 0.0014 | 109.0297 |
| 9 | disclosure | 57 | 0.0012 | 1 | 0.0000 | 103.9369 |
| 10 | federal | 74 | 0.0015 | 8 | 0.0001 | 101.8321 |
| 11 | bill | 50 | 0.0010 | 1 | 0.0000 | 90.3053 |
| 12 | Higher | 151 | 0.0031 | 69 | 0.0008 | 89.9071 |
| 13 | Congress | 71 | 0.0015 | 10 | 0.0001 | 89.6124 |
| 14 | NAS | 128 | 0.0027 | 55 | 0.0007 | 81.2197 |
| 15 | Department | 162 | 0.0034 | 88 | 0.0011 | 78.6717 |
| 16 | July | 56 | 0.0012 | 7 | 0.0001 | 73.6618 |
| 17 | Thousand | 37 | 0.0008 | 1 | 0.0000 | 65.1027 |
| 18 | universities | 295 | 0.0061 | 256 | 0.0031 | 61.5008 |
| 19 | influence | 86 | 0.0018 | 33 | 0.0004 | 60.722 |
| 20 | Institutes | 511 | 0.0106 | 541 | 0.0066 | 57.7417 |
| 21 | espionage | 34 | 0.0007 | 2 | 0.0000 | 53.8761 |
| 22 | Florida | 32 | 0.0007 | 2 | 0.0000 | 50.1427 |
| 23 | program | 105 | 0.0022 | 59 | 0.0007 | 48.7535 |
| 24 | transparency | 68 | 0.0014 | 26 | 0.0003 | 48.1654 |
| 25 | institutions | 79 | 0.0016 | 37 | 0.0005 | 45.7985 |
| 26 | American | 285 | 0.0059 | 274 | 0.0034 | 44.6958 |
| 27 | Texas | 37 | 0.0008 | 6 | 0.0001 | 44.2294 |
| 28 | Last | 42 | 0.0009 | 9 | 0.0001 | 44.1533 |
| 29 | Senate | 38 | 0.0008 | 7 | 0.0001 | 42.9941 |
| 30 | Security | 32 | 0.0007 | 4 | 0.0000 | 42.0925 |
| 31 | Defense | 25 | 0.0005 | 1 | 0.0000 | 42.0606 |
| 32 | conference | 56 | 0.0012 | 20 | 0.0002 | 42.0558 |
| 33 | require | 42 | 0.0009 | 10 | 0.0001 | 41.6983 |
| 34 | visa | 27 | 0.0006 | 2 | 0.0000 | 40.8778 |
| 35 | Scholars | 95 | 0.0020 | 57 | 0.0007 | 40.2158 |
| 36 | report | 123 | 0.0026 | 88 | 0.0011 | 38.9404 |
| 37 | researchers | 28 | 0.0006 | 3 | 0.0000 | 38.632 |
| 38 | Cancer | 23 | 0.0005 | 1 | 0.0000 | 38.2551 |
| 39 | military | 23 | 0.0005 | 1 | 0.0000 | 38.2551 |
| 40 | Subcommittee | 23 | 0.0005 | 1 | 0.0000 | 38.2551 |
| 41 | more | 132 | 0.0027 | 99 | 0.0012 | 38.1634 |
| 42 | close | 67 | 0.0014 | 32 | 0.0004 | 38.0072 |
| 43 | letter | 45 | 0.0009 | 14 | 0.0002 | 37.6147 |
| 44 | Mason | 21 | 0.0004 | 1 | 0.0000 | 34.4641 |
| 45 | protect | 30 | 0.0006 | 6 | 0.0001 | 32.652 |
| 46 | Agents | 23 | 0.0005 | 3 | 0.0000 | 29.8252 |
| 47 | public | 69 | 0.0014 | 42 | 0.0005 | 28.5937 |
| 48 | campuses | 42 | 0.0009 | 17 | 0.0002 | 28.2409 |
| 49 | Investigations | 20 | 0.0004 | 2 | 0.0000 | 28.138 |
| 50 | Justice | 20 | 0.0004 | 2 | 0.0000 | 28.138 |
| 51 | Technology | 20 | 0.0004 | 2 | 0.0000 | 28.138 |
| 52 | Christopher | 16 | 0.0003 | 1 | 0.0000 | 25.0713 |
| 53 | conditions | 16 | 0.0003 | 1 | 0.0000 | 25.0713 |
| 54 | Harvard | 16 | 0.0003 | 1 | 0.0000 | 25.0713 |
| 55 | disclose | 32 | 0.0007 | 11 | 0.0001 | 24.7911 |
| 56 | legislation | 18 | 0.0004 | 2 | 0.0000 | 24.5698 |
| 57 | George | 23 | 0.0005 | 5 | 0.0001 | 23.9984 |
| 58 | Threat | 23 | 0.0005 | 5 | 0.0001 | 23.9984 |
| 59 | address | 21 | 0.0004 | 4 | 0.0000 | 23.3959 |
| 60 | scientists | 15 | 0.0003 | 1 | 0.0000 | 23.212 |
| 61 | Information | 70 | 0.0015 | 49 | 0.0006 | 23.0644 |
| 62 | research | 92 | 0.0019 | 74 | 0.0009 | 22.9582 |
| 63 | Association | 81 | 0.0017 | 62 | 0.0008 | 22.4728 |
| 64 | year | 59 | 0.0012 | 38 | 0.0005 | 22.4001 |
| 65 | source | 20 | 0.0004 | 4 | 0.0000 | 21.768 |
| 66 | Boston | 14 | 0.0003 | 1 | 0.0000 | 21.3609 |
| 67 | company | 14 | 0.0003 | 1 | 0.0000 | 21.3609 |
| 68 | Charged | 18 | 0.0004 | 3 | 0.0000 | 21.2747 |
| 69 | million | 24 | 0.0005 | 7 | 0.0001 | 20.9942 |
| 70 | should | 127 | 0.0026 | 120 | 0.0015 | 20.9839 |
| 71 | greater | 19 | 0.0004 | 4 | 0.0000 | 20.1568 |
| 72 | Download | 17 | 0.0004 | 3 | 0.0000 | 19.6067 |
| 73 | donations | 13 | 0.0003 | 1 | 0.0000 | 19.5195 |
| 74 | donors | 13 | 0.0003 | 1 | 0.0000 | 19.5195 |
| 75 | government-funded | 13 | 0.0003 | 1 | 0.0000 | 19.5195 |
| 76 | Illegal | 13 | 0.0003 | 1 | 0.0000 | 19.5195 |
| 77 | FALSE | 15 | 0.0003 | 2 | 0.0000 | 19.3046 |
| 78 | lab | 15 | 0.0003 | 2 | 0.0000 | 19.3046 |
| 79 | investigation | 21 | 0.0004 | 6 | 0.0001 | 18.6281 |
| 80 | sources | 21 | 0.0004 | 6 | 0.0001 | 18.6281 |
| 81 | pleased | 18 | 0.0004 | 4 | 0.0000 | 18.5639 |
| 82 | https | 47 | 0.0010 | 30 | 0.0004 | 18.1027 |
| 83 | Virginia | 16 | 0.0003 | 3 | 0.0000 | 17.9565 |
| 84 | efforts | 28 | 0.0006 | 12 | 0.0001 | 17.813 |
| 85 | purpose | 19 | 0.0004 | 5 | 0.0001 | 17.7734 |
| 86 | urge | 19 | 0.0004 | 5 | 0.0001 | 17.7734 |
| 87 | disclosed | 12 | 0.0002 | 1 | 0.0000 | 17.689 |
| 88 | existing | 14 | 0.0003 | 2 | 0.0000 | 17.5786 |
| 89 | United | 72 | 0.0015 | 60 | 0.0007 | 16.5695 |
| 90 | threats | 15 | 0.0003 | 3 | 0.0000 | 16.326 |
| 91 | test | 22 | 0.0005 | 8 | 0.0001 | 16.2747 |
| 92 | Review | 30 | 0.0006 | 15 | 0.0002 | 16.1442 |
| 93 | programs | 61 | 0.0013 | 48 | 0.0006 | 15.9696 |
| 94 | scientific | 11 | 0.0002 | 1 | 0.0000 | 15.8714 |
| 95 | changes | 13 | 0.0003 | 2 | 0.0000 | 15.8706 |
| 96 | complaint | 19 | 0.0004 | 6 | 0.0001 | 15.7094 |
| 97 | board | 140 | 0.0029 | 149 | 0.0018 | 15.5065 |
| 98 | States | 73 | 0.0015 | 63 | 0.0008 | 15.4317 |
| 99 | Secretary | 14 | 0.0003 | 3 | 0.0000 | 14.7178 |
| 100 | plan | 22 | 0.0005 | 9 | 0.0000 | 14.6448 |
